# Assessment and Management of Cardiovascular Risk Factors Among US Veterans With Prostate Cancer

**DOI:** 10.1001/jamanetworkopen.2021.0070

**Published:** 2021-02-24

**Authors:** Lova Sun, Ravi B. Parikh, Rebecca A. Hubbard, John Cashy, Samuel U. Takvorian, David J. Vaughn, Kyle W. Robinson, Vivek Narayan, Bonnie Ky

**Affiliations:** 1 Division of Hematology/Oncology, Department of Medicine, Perelman School of Medicine, University of Pennsylvania, Philadelphia; 2 Abramson Cancer Center, Perelman School of Medicine, University of Pennsylvania, Philadelphia; 3 Department of Hematology/Oncology, Corporal Michael J. Crescenz Veterans Affairs Medical Center, Philadelphia, Pennsylvania; 4 Department of Medical Ethics and Health Policy, Perelman School of Medicine, University of Pennsylvania, Philadelphia; 5 Center for Health Equity Research and Promotion, Veterans Affairs Pittsburgh Healthcare System, Pittsburgh, Pennsylvania; 6 Department of Biostatistics, Epidemiology & Informatics, Perelman School of Medicine, University of Pennsylvania, Philadelphia; 7 Division of Cardiology, Department of Medicine, Perelman School of Medicine, University of Pennsylvania, Philadelphia

## Abstract

**Importance:**

Cardiovascular disease is a leading cause of mortality in patients with prostate cancer, and androgen deprivation therapy (ADT) may worsen cardiovascular risk. Adherence to guideline-recommended assessment and management of cardiovascular risk factors (CVRFs) in patients initiating ADT is unknown.

**Objective:**

To describe CVRF assessment and management in men with prostate cancer initiating ADT and overall.

**Design, Setting, and Participants:**

A cross-sectional analysis of 90 494 men treated within the US Veterans Health Administration diagnosed with prostate cancer between January 1, 2010, and December 31, 2017, was conducted. Participants included men with a history of atherosclerotic cardiovascular disease (ASCVD), and treatment with ADT within 1 year of diagnosis. Data analysis was conducted from September 10, 2019, to July 1, 2020.

**Main Outcomes and Measures:**

Rates of comprehensive CVRF assessment, uncontrolled CVRFs, and untreated CVRFs. Comprehensive CVRF assessment was defined as recorded measures for blood pressure, cholesterol, and glucose levels; CVRF control as blood pressure lower than 140/90 mm Hg, low-density lipoprotein cholesterol 130 mg/dL, and hemoglobin A_1c_ less than 7%; and CVRF treatment as receipt of cardiac risk–reducing medications. Multivariable risk difference regression assessed the association between ASCVD and initiation of ADT and these outcomes.

**Results:**

Of 90 494 veterans, median age was 66 years (interquartile range, 62-70 years); and 22 700 men (25.1%) received ADT. Overall, 68.1% (95% CI, 67.8%-68.3%) of the men received comprehensive CVRF assessment; 54.1% (95% CI. 53.7%-54.4%) of those assessed had uncontrolled CVRFs, and 29.6% (95% CI, 29.2%-30.0%) of those with uncontrolled CVRFs were not receiving corresponding cardiac risk–reducing medication. Compared with the reference group of patients without ASCVD not receiving ADT, patients with ASCVD not receiving ADT had a 10.4% (95% CI, 9.5%-11.3%) higher probability of comprehensive CVRF assessment, 4.0% (95% CI, 2.9%-5.1%) lower risk of uncontrolled CVRFs, and 22.2% (95% CI, 21.1%-23.3%) lower risk of untreated CVRFs. Similar differences were observed in patients with ASCVD receiving ADT. In contrast, patients without ASCVD receiving ADT had only a 3.0% (95% CI, 2.1%-3.9%) higher probability of comprehensive CVRF assessment, 2.6% (95% CI, 1.6%-3.5%) higher risk of uncontrolled CVRFs, and 5.4% (95% CI, 4.2%-6.6%) lower risk of untreated CVRFs.

**Conclusions and Relevance:**

These findings suggest that veterans with prostate cancer had a high rate of underassessed and undertreated CVRFs, and ADT initiation was not associated with substantial improvements in CVRF assessment or management. These findings highlight gaps in care and the need for interventions to improve CVRF mitigation in this population.

## Key Points

**Question:**

Are cardiovascular risk factors assessed and appropriately managed in patients with prostate cancer initiating androgen deprivation therapy?

**Findings:**

In this cross-sectional analysis of 90 494 US veterans with prostate cancer, 68.1% received comprehensive cardiovascular risk factor assessment and 54.1% had uncontrolled risk factors; of these, 29.6% were not receiving risk-reducing medication. Patients with known atherosclerotic cardiovascular disease had improved cardiovascular risk factor assessment, control, and treatment; however, androgen deprivation therapy initiation was not associated with meaningful differences in these outcomes.

**Meaning:**

In this study, veterans with prostate cancer, including those initiating androgen deprivation therapy, appeared to have a high burden of underassessed and undertreated cardiovascular risk factors.

## Teaser

This cross-sectional study examines the assessment and management of cardiovascular risk factors among men initiating androgen deprivation therapy for prostate cancer.

## Introduction

Improvements in prostate cancer survival have led to an increasing emphasis on competing health risks and survivorship care.^1^ In particular, cardiovascular risk factors (CVRFs) and atherosclerotic cardiovascular disease (ASCVD) are prevalent^2,3^ and leading causes of mortality in men with prostate cancer.^4,5,6^ Increasing evidence points to overlapping biologic risk factors for cancer and cardiovascular disease,^7^ and mitigation of modifiable CVRFs in patients with cancer represents an opportunity to optimize survival and survivorship outcomes.

Androgen deprivation therapy (ADT), although a standard and effective treatment for prostate cancer, has been associated with an increased risk of diabetes, metabolic syndrome, and ASCVD.^8,9,10,11^ This risk may be most pronounced within the first 6 months of therapy and in patients with a history of ASCVD.^12^ In addition, men receiving ADT may already be at higher cardiac risk because shared risk factors, such as smoking and obesity, are associated with both cardiovascular disease and high-risk for prostate cancer.^2,3,13^

In 2010, increased recognition of the potential cardiovascular toxic effects of ADT led to the addition of US Food and Drug Administration warnings to gonadotropin-releasing hormone agonist labels, as well as a joint scientific statement from the American Heart Association, American Cancer Society, and American Urologic Association, which recommended assessment for CVRFs, including blood pressure, lipid profile, and glucose level, in patients initiating ADT.^14^ Expert consensus groups also recommend the ABCDE (awareness/aspirin, blood pressure, cholesterol/cigarettes, diabetes, and exercise) method to mitigate cardiovascular risk in patients undergoing ADT.^15^ However, to our knowledge, no studies to date have evaluated population-based adherence to CVRF screening and management in patients with prostate cancer, including those undergoing ADT.

To address this knowledge gap, we performed a cross-sectional analysis of US veterans diagnosed with prostate cancer from January 1, 2010, to December 31, 2017, to characterize rates and determinants of baseline CVRF assessment and management. US veterans have a high burden of both prostate cancer and cardiac disease,^16,17,18^ with more than 15 000 cases of prostate cancer diagnosed annually within the Veterans Affairs (VA) network.^19^ We hypothesized that a significant proportion of veterans with prostate cancer do not receive CVRF assessment and management concordant with consensus recommendations and that uncontrolled modifiable CVRFs are prevalent in this population. Given the known adverse cardiovascular effects of ADT, particularly in those with underlying ASCVD, we also sought to examine the association of ADT initiation with CVRF assessment and management in those with and without a history of ASCVD.

## Methods

### Data Source and Study Population

Veterans with histologically confirmed prostate cancer diagnosed from January 1, 2010, to December 31, 2017, were identified from the VA Corporate Data Warehouse. This time period was chosen to capture a contemporary patient cohort following the publication of ADT cardiovascular risk consensus scientific statements^14^ and cardiovascular guidelines.^20,21,22,23,24^ Patient-level information, including demographic and prostate cancer–specific parameters, laboratory results, vital signs, medications dispensed from VA pharmacies, and diagnosis and procedure codes from inpatient and outpatient visits, was obtained through the VA national electronic health records. This study was approved and informed consent requirement was waived by the institutional review board at the Corporal Michael J. Crescenz VA Medical Center in Philadelphia, Pennsylvania. This study followed the Strengthening the Reporting of Observational Studies in Epidemiology (STROBE) reporting guideline.

Patients were categorized according to whether they had previous ASCVD (defined as 1 inpatient or 2 outpatient visits with *International Classification of Diseases, Ninth Revision* and *International Statistical Classification of Diseases, 10th Revision* codes in the year before diagnosis for coronary artery disease, stroke, or peripheral vascular disease),^25,26,27^ and whether they received ADT within a year of diagnosis (defined as either a procedure code or injection date for ADT)^28^ (eTable 1 in the Supplement). Of these 4 groups (no history of ASCVD, not receiving ADT [ASCVD−/ADT−]; no history of ASCVD, receiving ADT [ASCVD−/ADT+]; history of ASCVD, not receiving ADT [ASCVD+/ADT−], history of ASCVD, receiving ADT [ASCVD+/ADT+]), patients without prior ASCVD not receiving ADT (ASCVD−/ADT−) served as the reference group for regression analyses.

Each patient’s 18-month study period was defined as the 12 months before to 6 months following the baseline date, which was the first ADT injection date if treated with ADT or the date of prostate cancer diagnosis if not treated with ADT. The 6-month postbaseline time window was chosen to allow adequate time to capture CVRF assessment and management decisions prompted by the decision to initiate ADT. To restrict our analysis to patients receiving comprehensive care within the VA system in whom data on CVRF assessment and management could be accurately assessed, patients were excluded if they did not have a documented VA primary care physician visit during the study period.^29,30^ Patients were also excluded if their ADT administration status could not be confirmed owing to a recorded ADT injection date without a corresponding procedure code.

### Outcomes

Comprehensive CVRF assessment was defined as having at least 1 recorded measure during the 18-month study period for all of the following measures, per the American Heart Association, American Cancer Society, and American Urologic Association recommendations^3,14^: blood pressure (both systolic and diastolic), cholesterol level (either low-density lipoprotein or total cholesterol), and blood glucose level (either hemoglobin A_1c_, fasting glucose, or glucose tolerance test) (eTable 1 in the Supplement). Random nonfasting glucose level alone (eg, glucose level determined as part of a metabolic panel) was not counted as glucose assessment.^24^

Among patients with recorded CVRF measures, we characterized the proportion of patients with uncontrolled blood pressure (systolic ≥140 mm Hg or diastolic ≥90 mm Hg^31^), cholesterol levels (low-density lipoprotein cholesterol ≥130 mg/dL or total cholesterol ≥240 mg/dL [to convert to millimoles per liter, multiply by 0.0259]), and glucose levels (hemoglobin A_1c_≥7% [to convert to proportion of total hemoglobin, multiply by 0.01] or fasting glucose ≥126 mg/dL [to convert to millimoles per liter, multiply by 0.0555]^32^). These CVRF control thresholds reflect definitions from consensus guidelines of professional societies, including the American Heart Association, American College of Cardiology, American Diabetes Association, and American College of Physicians,^20,21,22,23,24^ as well as those used in earlier large, retrospective database studies.^33^ For baseline systolic and diastolic blood pressure, we used the median of all measures recorded in the year before the baseline date to avoid capturing the potential early effects of ADT administration. If multiple recorded laboratory measures were available for a patient during the study period, we used the most proximal result before the baseline date or the most proximal result after the baseline date if no prebaseline result was available. We then characterized the proportion of patients with uncontrolled blood pressure, cholesterol, or glucose levels who were not prescribed a corresponding risk-reducing cardiac medication (eTable 1 in the Supplement).

### Statistical Analysis

Data analysis was conducted from September 10, 2019, to July 1, 2020. Patient demographic and disease characteristics were compared between ADT-treated and untreated groups by the Mann-Whitney test for continuous variables and χ^2^ test for categorical variables. Point estimates and 95% CIs were calculated for the following outcomes: (1) proportion of patients with comprehensive CVRF assessment, (2) proportion of patients with CVRF assessment with uncontrolled CVRFs, and (3) proportion of patients with uncontrolled CVRFs not prescribed a cardiovascular risk–reducing medication (ie, untreated). We used risk difference regression analyses with robust SEs to identify patient characteristics associated with the probability of comprehensive CVRF assessment and uncontrolled and untreated CVRFs.^34^ Risk difference coefficients were interpreted as the additive percent difference in risk of the outcome relative to the reference group. Predictor variables included in these models were age, race, national poverty index based on neighborhood mapping,^35^ disease stage (based on tumor stage, prostate-specific antigen, and Gleason score), and baseline year, as well as our main covariates of interest (treatment with ADT and prior ASCVD). We then examined the interaction between ASCVD (yes/no) and ADT treatment (yes/no) to determine the association between ADT and CVRF assessment and how this association might differ based on preexisting ASCVD status. In multivariable analyses, observations with a missing value for any covariate were excluded.

Because cardiovascular risk mitigation may have been deprioritized in patients with advanced prostate cancer at higher risk of cancer-specific mortality, we performed sensitivity analyses for each outcome excluding patients with metastatic or node-positive cancer. Statistical significance was defined using a 2-sided α level of .05 for all analyses, including interaction analyses. Stata, version 15 (StataCorp LLC) was used for all analyses.

## Results

### Patient Cohort

**Figure 1.**
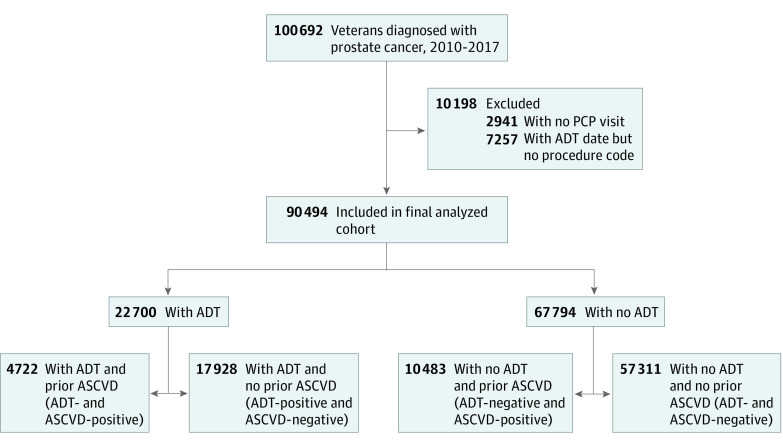
Study Population Flow ADT indicates androgen deprivation therapy; ASCVD, atherosclerotic cardiovascular disease; and PCP, primary care physician.

We identified 100 692 patients diagnosed with prostate cancer from 2010 to 2017, of whom 90 494 were included in our primary analyses (Figure 1). Included patients had a median age of 66 years (interquartile range, 62-70 years); 67.3% were White, and 66.0% were current or former smokers. Patients excluded owing to lack of a primary care physician visit or lack of confirmed ADT status had similar demographic and disease parameters compared with the analysis cohort, but had higher proportions of missing data (eTable 2 in the Supplement), suggesting a higher likelihood of care outside of the VA system.

The final analysis cohort consisted of 22 700 patients receiving ADT and 67 794 patients not receiving ADT (Figure 1). The annual proportion of patients initiating ADT remained relatively stable (range, 23.3%-27.8%) from 2010 to 2017. Patients receiving ADT were more likely to be older (median age, 67 [interquartile range, 63-74] vs 65 [interquartile range, 61-69] years), have a history of ASCVD (21.0% vs 15.5%), and have metastatic or node-positive disease (23.2% vs 1.6%) (Table 1). Of the entire cohort, 78.1% were overweight or obese (body mass index ≥25 [calculated as weight in kilograms divided by height in meters squared]).

**Table 1.** Baseline Demographic Characteristics, Veterans Diagnosed With Prostate Cancer 2010-2017

Characteristic	Treated with ADT, No. (%)^a^	
	Yes (n = 22 700)	No (n = 67 794)
Age, median (IQR), y	67 (63-74)	65 (61-69)
Race		
White	14 315 (66.0)	43 857 (67.7)
African American	6864 (31.7)	19 346 (29.9)
Other^b^	507 (2.3)	1536 (2.4)
Year category		
2010-2011	6262 (27.6)	19 948 (29.4)
2012-2013	5430 (23.9)	17 069 (25.2)
2014-2015	5445 (24.0)	15 863 (23.4)
2016-2017	5563 (24.5)	14 914 (22.0)
No. of PCP visits over study period, median (IQR)	5 (4-8)	5 (3-7)
Poverty index above median	14 501 (64.3)	41 975 (62.3)
Prior ASCVD	4772 (21.0)	10 483 (15.5)
Smoking status		
Never	6027 (33.9)	17 894 (34.1)
Current	6286 (35.3)	18 312 (34.9)
Former	5485 (30.8)	16 307 (31.1)
BMI, median (IQR)	28.4 (24.9,32.5)	28.7 (25.6,32.4)
Overweight (BMI ≥25)	15 161 (74.3)	47 852 (79.4)
Obese (BMI >30)	7963 (39.0)	24 058 (39.9)
Disease stage		
Low risk	721 (3.3)	25 591 (39.0)
Intermediate risk	5744 (26.2)	20 221 (30.8)
High risk	10 347 (47.3)	18 740 (28.6)
Metastatic or node positive	5079 (23.2)	1048 (1.6)
PSA, ng/mL		
<10	10 117 (47.2)	52 806 (82.7)
10-20	4582 (21.4)	8067 (12.6)
≥20	6731 (31.4)	2944 (4.6)
Gleason score		
≤6	1326 (6.5)	28 226 (46.0)
7	8159 (39.9)	28 170 (45.9)
8-10	10 986 (53.7)	5026 (8.2)
T category		
T1-T2a	12 431 (64.0)	43 419 (72.5)
T2b	1240 (6.4)	1303 (2.2)
≥T2c	5750 (29.6)	15 142 (25.3)

Abbreviations: ADT, androgen deprivation therapy; ASCVD, atherosclerotic cardiovascular disease; BMI, body mass index (calculated as weight in kilograms divided by height in meters squared); IQR, interquartile range; PCP, primary care physician; PSA, prostate specific antigen.

SI conversion: To convert PSA to micrograms per liter, multiply by 1.

^a^ Unless otherwise indicated, data are expressed as number (percentage) of patients with recorded data.

^b^ Other includes American Indian or Alaska Native, Asian, Native Hawaiian or other Pacific Islander, and multiple categories.

### CVRF Assessment

**Figure 2.**
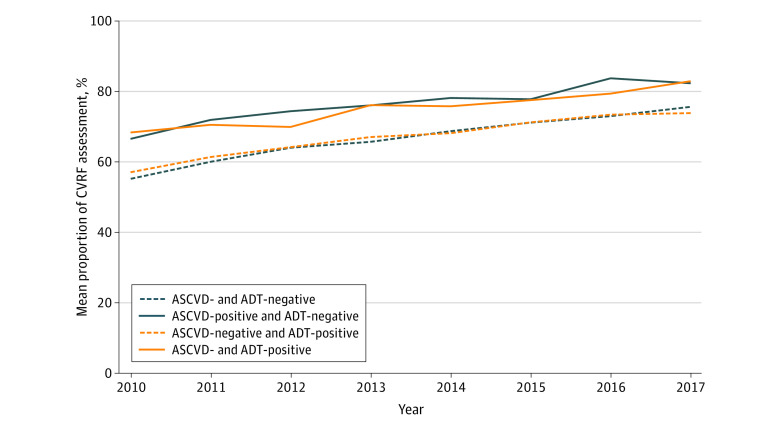
Proportion of Comprehensive Cardiovascular Risk Factor (CVRF) Assessment Over Time, According to Androgen Deprivation Therapy (ADT) and Atherosclerotic Cardiovascular Disease (ASCVD) Group Patients were categorized into 4 groups based on history of ASCVD and receipt of ADT within a year of diagnosis. Yearly unadjusted proportions of patients with recorded measurements for all 3 CVRFs (blood pressure, glucose, and cholesterol levels) within the study period are shown.

Overall, only 68.1% (95% CI, 67.8-68.3) of veterans received comprehensive CVRF assessment, as defined by recorded measures for blood pressure, lipid levels, and glucose level. Rates of comprehensive CVRF assessment increased steadily over time, from 57.9% (95% CI, 57.0%-58.7%) in 2010 to 76.8% (95% CI, 76.0%-77.6%) in 2017 (Figure 2), mostly secondary to improved glucose screening (eFigure in the Supplement). White race was associated with a 5.7% (95% CI, 5.0%-6.4%) lower probability of comprehensive CVRF assessment compared with non-White race, and metastatic disease was associated with a 7.4% lower (95% CI, 5.8%-8.9%) probability of comprehensive assessment compared with low-risk localized disease (Table 2).

**Table 2.** Adjusted Risk Differences for Probability of Comprehensive CVRF Assessment

Comprehensive CVRF assessment	Adjusted risk difference, % (95% CI)^a^	*P* value
Race		
White	−5.7 (−6.4 to −5.0)	<.001
Non White	1 [Reference]	
Age, y		
≤60	1 [Reference]	
61-65	2.2 (13-3.1)	<.001
66-70	2.4 (1.5-3.4)	<.001
71-75	1.5 (0.3-2.7)	.01
>75	−4.3 (−5.6 to −2.9)	<.001
National Poverty Index above median	−0.6 (−1.2 to 0.1)	.08
Disease stage		
Low risk	1 [Reference]	
Intermediate risk	0.0 (−0.8 to 0.8)	.96
High risk	−1.3 (−2.1 to −0.5)	.002
Metastatic or node positive	−7.4 (−8.9 to −5.8)	<.001
Baseline year		
2010-2011	1 [Reference]	
2012-2013	6.3 (5.4-7.2)	<.001
2014-2015	11.1 (10.2-12.0)	<.001
2016-2017	15.2 (14.4-16.1)	<.001
ASCVD/ADT status^b^		
ASCVD−/ADT−	1 [Reference]	
ASCVD+/ADT−	10.4 (9.5-11.3)	<.001
ASCVD+/ADT+	12.3 (10.9-13.7)	<.001
ASCVD−/ADT+	3.0 (2.1-3.9)	<.001

Abbreviations: ADT, androgen deprivation therapy; ASCVD, atherosclerotic cardiovascular disease; CVRF, cardiovascular risk factor.

^a^ Risk difference coefficients are interpreted as absolute percent difference in the probability of outcome. All coefficients were adjusted for other covariates in the model.

^b^ ASCVD−/ADT− indicates no history of ASCVD, not receiving ADT; ASCVD+/ADT−, history of ASCVD, not receiving ADT; ASCVD+/ADT+, history of ASCVD, receiving ADT; and ASCVD−/ADT+, no history of ASCVD, receiving ADT.

In multivariable regression analyses, in patients with known ASCVD, the adjusted proportion of comprehensive CVRF assessment was 78.2% (95% CI, 76.9%-79.5%) in ADT-treated patients and 76.2% (95% CI, 75.4%-77.1%) in ADT-untreated patients. In comparison, patients without ASCVD had lower adjusted proportions of comprehensive CVRF assessment: 68.8% (95% CI, 68.1%-69.6%) in ADT-treated patients and 65.8% (95% CI, 65.4%-66.3%) in ADT-untreated patients. Compared with the ASCVD−/ADT− reference group, ASCVD+/ADT− patients had a 10.4% (95% CI, 9.5%-11.3%) higher probability of comprehensive CVRF assessment, and ASCVD+/ADT+ patients had a similar 12.3% (95% CI, 10.9%-13.7%) higher probability of comprehensive CVRF assessment (Table 2). eTable 3 in the Supplement provides unadjusted results. In contrast, in ASCVD−/ADT+ patients, there was only a 3.0% (95% CI, 2.1%-3.9%) higher probability of comprehensive assessment compared with the ASCVD−/ADT− reference group. These results suggest that comprehensive CVRF assessment was primarily associated with ASCVD status rather than planned ADT. The interaction between ASCVD status and ADT initiation did not meet our a priori threshold for statistical significance (*P* = .23).

### CVRF Control

Of all veterans with CVRF assessment, 35.7% (95% CI, 35.3%-36.0%) had uncontrolled blood pressure, 19.8% (95% CI, 19.5%-20.0%) had uncontrolled cholesterol levels, and 19.1% (95% CI, 18.8%-19.4%) had uncontrolled glucose levels. Overall, 54.1% (95% CI, 53.7%-54.4%) had at least 1 uncontrolled CVRF. In adjusted analysis, compared with the ASCVD−/ADT− reference group, patients with ASCVD+/ADT− had a 4.0% (95% CI, 2.9%-5.1%) lower absolute risk of having at least 1 uncontrolled CVRF. Similarly, patients with ASCVD+/ADT+ had a 2.2% (95% CI, 0.6%-3.8%) lower risk of having uncontrolled CVRFs. In contrast, there was a 2.6% (95% CI, 1.6%-3.5%) greater risk of uncontrolled CVRFs in patients with ASCVD−/ADT+ compared with the reference group. Adjusted risk differences associated with ADT and ASCVD status for uncontrolled blood pressure, cholesterol, and glucose levels are detailed in Table 3. Unadjusted results are listed in eTable 3 in the Supplement. These differences are not likely to reflect the biologic effects of ADT, given that all blood pressure measurements and more than 95% of cholesterol and glucose level measures were obtained before the baseline date. The risk of uncontrolled blood pressure differed according to a whether the patient had a history of ASCVD (interaction *P* = .004). There was no significant interaction by ASCVD on the association between ADT and uncontrolled cholesterol level (*P* = .10), glucose (*P* = .84) level, or the composite outcome of any uncontrolled CVRF (*P* = .49).

**Table 3.** Adjusted Proportions and Risk Differences for Uncontrolled and Untreated CVRFs

CVRF	Uncontrolled CVRF					Untreated CVRF				
	No. with recorded value	Adjusted, % (95% CI)				No. uncontrolled	Adjusted, % (95% CI)			
		Proportion uncontrolled, ASCVD−/ADT− [reference]^a^	Risk difference^b^				Proportion untreated, ASCVD−/ADT− [reference]^c^	Risk difference^b^		
			ASCVD+/ADT-	ASCVD+/ADT+	ASCVD-/ADT+			ASCVD+/ADT−	ASCVD+/ADT+	ASCVD−/ADT+
Blood pressure	89 572	35.8 (35.4 to 36.3)	−4.6 (−5.6 to −3.6)	−4.7 (−6.2 to −3.1)	2.6 (1.7 to 3.6)	31 960	24.5 (23.8 to 25.1)	−15.9 (−17.1 to −14.7)	−18.1 (−19.7 to −16.4)	−4.3 (−5.5 to −3.0)
Cholesterol	85 243	21.7 (21.3 to 22.1)	−8.9 (−9.7 to −8.2)	−9.5 (−10.5 to −8.4)	−1.6 (−2.4 to −0.8)	16 879	50.8 (49.9 to 51.8)	−32.0 (−34.4 to −29.5)	−29.4 (−33.6 to −25.2)	−2.5 (−4.7 to −0.3)
Glucose	63 292	16.7 (16.3 to 17.1)	8.3 (7.2 to 9.4)	11.3 (9.7 to 12.9)	3.2 (2.3 to 4.2)	12 098	9.4 (8.7 to 10.2)	−3.1 (−4.4 to −1.8)	−3.6 (−5.5 to −1.6)	−2.7 (−4.1 to −1.3)
Any CVRF	90 060	54.1 (53.7 to 54.6)	−4.0 (−5.1 to −2.9)	−2.2 (−3.8 to −0.6)	2.6 (1.6 to 3.5)	48 683	34.1 (33.5 to 34.6)	−22.2 (−23.3 to −21.1)	−23.3 (−24.9 to −21.8)	−5.4 (−6.6 to −4.2)

Abbreviations: ADT, androgen deprivation therapy; ASCVD, atherosclerotic cardiovascular disease; CVRF, cardiovascular risk factor.

^a^ Adjusted proportion of patients in the reference group (No ASCVD, No ADT) with recorded measurements who have uncontrolled CVRFs.

^b^ Adjusted for race, age, poverty index, disease stage, and year. Risk difference coefficients are interpreted as absolute percent change in the risk of outcome.

^c^ Adjusted proportion of patients in the reference group (No ASCVD, No ADT) with uncontrolled CVRFs who are not on a corresponding risk-reducing medication.

### CVRF Treatment

Overall, 21.3% (95% CI, 20.9%-21.8%) of patients with uncontrolled blood pressure were not receiving antihypertensive medications; 47.6% (95% CI, 46.8%-48.3%) of patients with uncontrolled cholesterol levels were not receiving lipid-lowering therapy; and 8.1% (95% CI, 7.6%-8.6%) of patients with uncontrolled blood glucose levels were not receiving antihyperglycemic therapy. Of all patients with uncontrolled CVRFs, 29.6% (95% CI, 29.2%-30.0%) were not receiving a corresponding risk-reducing medication.

Patients with ASCVD+/ADT− had a 22.2% (95% CI, 21.1%-23.3%) lower risk of untreated CVRFs (ie, not receiving cardiac risk–reducing medication) compared with the reference group (ASCVD−/ADT−); this difference was similar in the ASCVD+/ADT+ group, who had a 23.3% (95% CI, 21.8%-24.9%) lower risk of untreated CVRFs compared with the reference group. In contrast, in patients with ASCVD−/ADT+, there was only a 5.4% (95% CI, 4.2%-6.6%) lower risk of untreated CVRFs. Adjusted risk differences associated with ADT and ASCVD status for untreated BP, cholesterol, and glucose levels are reported in Table 3. Unadjusted results are listed in eTable 3 in the Supplement. These findings suggest that ASCVD history, but not ADT, was associated with closer cardiovascular risk management. The risks of untreated BP, untreated cholesterol levels, and any untreated CVRF among those receiving ADT differed according to a history of ASCVD (interaction blood pressure, *P* = .047; cholesterol level, *P* = .045; and untreated CVRF, *P* < .001 for interaction); this interaction was not significant for untreated glucose levels (*P* = .07).

### Sensitivity Analysis

In sensitivity analyses excluding patients with metastatic or node-positive disease who composed 7.0% of our cohort, we found similar rates of comprehensive CVRF assessment (68.6%; 95% CI, 68.3%-68.9%), uncontrolled CVRFs (54.2%; 95% CI, 53.9%-54.5%), and untreated CVRFs (29.4%; 95% CI, 29.0%-29.8%). Estimates of the association between ASCVD and ADT and these outcomes were also similar to those in the entire cohort (eTable 4 in the Supplement).

## Discussion

We conducted a nationwide population-based study of CVRF assessment and management in patients with prostate cancer, in whom cardiovascular disease is a leading cause of morbidity and mortality.^6^ Our analysis of more than 90 000 veterans with prostate cancer diagnosed from 2010 to 2017 showed that, although CVRF assessment improved over time, largely reflective of a positive change in hemoglobin A_1c_ screening rates within the VA system, rates of incomplete assessment remained high, with more than 1 in 5 veterans not receiving comprehensive CVRF assessment even in recent years. We also found a high burden of modifiable risk factors at baseline: more than three-quarters were overweight or obese; more than half had uncontrolled blood pressure, cholesterol, and/or glucose levels; and more than a quarter of these patients were not receiving corresponding risk-reducing medications.

Our findings expand on those of earlier studies reporting adverse CVRF profiles and suboptimal risk management in patients with prostate cancer. In the HERO trial, which compared relugolix with leuprolide in men with advanced prostate cancer, more than 90% of patients had at least 1 CVRF, despite exclusion of patients with recent major adverse cardiovascular events.^36^ In the RADICAL-PC study of a multimodal approach to modifying cardiovascular risk factors in men with prostate cancer,^37^ two-thirds of enrolled patients had high cardiovascular risk by the Framingham score.^38^ A prospective multi-institutional study of 103 patients receiving ADT with radiotherapy found that only 63% received CVRF monitoring concordant with American Heart Association guidelines, 24% had uncontrolled blood glucose levels, and 22% had uncontrolled cholesterol levels after 1 year.^3^

To our knowledge, this study represents the first US nationwide systematic analysis of CVRF assessment and treatment patterns in a large population of patients with prostate cancer and highlights important gaps therein. Although cardiac risk was assessed and managed more closely in veterans with a history of ASCVD, initiation of ADT was not associated with meaningfully higher rates of CVRF assessment or treatment, despite increasing awareness and consensus statements regarding the cardiovascular effects of ADT. In particular, although patients with known ASCVD treated with ADT may represent an especially vulnerable group at high risk for cardiac events, we did not observe a corresponding increase in cardiac risk mitigation intensity in this group.

Several factors may help explain the lack of meaningful association between ADT initiation and cardiac risk mitigation. First, this finding may reflect ongoing perceived controversy regarding the causal relationship between ADT and adverse cardiovascular events, given that some studies have not shown an association.^39,40^ Second, patients treated with ADT are more likely to experience symptomatic adverse effects, including hot flashes and decreased libido, which may require increased clinical time at the cost of attention to more asymptomatic but clinically important toxic reactions, including cardiometabolic effects. Because monitoring and mitigation of CVRFs are essential to improving survivorship care in patients with prostate cancer, most of whom are treated with curative intent and have prolonged life expectancies,^6,15,41^ our findings underscore the need for improved clinician and patient education, as well as interventions to optimize cardiac risk management. These efforts may emphasize multidisciplinary collaboration between oncologists, radiation oncologists, urologists, primary care physicians, and cardiologists.^42^

### Limitations

This study has limitations. Definitions of ASCVD status and CVRF assessment and control were based on review of guidelines and codes used in previously published literature,^25,27,43^ but misclassification remains possible given reliance on electronic medical record data. Furthermore, standards for CVRF control and treatment have changed over time and vary across patient comorbidity status. To address this heterogeneity and potential for misclassification, we restricted our analysis to a contemporary cohort managed under recent cardiac risk recommendations and following the promulgation of cardiovascular warnings associated with ADT, stratified our analysis by prior ASCVD status, and used relatively lenient CVRF control thresholds to reflect an older cancer population.

Given the care pattern variations between the VA and civilian settings, the generalizability of these findings to a nonveteran population is unclear. In addition, veterans excluded from the analyzed cohort owing to lack of VA primary care physician visit or unconfirmed ADT administration may have differed systematically from the analyzed population; however, these patients composed less than 10% of the study population. Although uncaptured CVRF measurements or treatment performed outside of the VA system remain possible, we required a VA primary care physician visit to mitigate this potential bias. Despite restricting our analysis to patients most likely to have consistent follow-up care within the VA system, a health care system that often outperforms non-VA settings in quality-of-care metrics,^44,45^ we still found significant rates of suboptimal cardiac risk assessment and mitigation.

## Conclusions

This cross-sectional analysis of more than 90 000 US veterans with prostate cancer over the past decade suggests progress, but also important areas of unmet need, in the assessment and management of cardiovascular risk. Whereas previous ASCVD was associated with consistently improved CVRF assessment, control, and risk-reducing medication therapy, the initiation of ADT was not associated with clinically meaningful differences. These findings suggest the need for closer clinical attention and education, as well as innovative tools and interventions, to improve the stratification and mitigation of cardiac risk in patients with prostate cancer.

## Notes

Supplement. **eTable 1.** Coding Definitions **eTable 2.** Demographic Parameters, Excluded Patients **eFigure.** Rates of Comprehensive CVRF Assessment **eTable 3.** Unadjusted Proportions and Risk Differences Associated With ASCVD/ADT Status **eTable 4.** Sensitivity Analysis: Adjusted Proportions and Risk Differences in Patients With Localized Disease Only
