# Supplementary material for: Assessment and Management of Cardiovascular Risk Factors Among US Veterans With Prostate Cancer
Source: JAMA Netw Open. 2021 Feb 24;4(2):e210070. doi: 10.1001/jamanetworkopen.2021.0070 (PMC7905496; doi:10.1001/jamanetworkopen.2021.0070)
Supplement: Supplement. — eTable 1. Coding Definitions eTable 2. Demographic Parameters, Excluded Patients eFigure. Rates of Comprehensive CVRF Assessment eTable 3. Unadjusted Proportions and Risk Differences Associated With ASCVD/ADT Status eTable 4. Sensitivity Analysis: Adjusted Proportions and Risk Differences in Patients With Localized Disease Only [file jamanetwopen-e210070-s001.pdf]

Supplementary Online Content

Sun L, Parikh RB, Hubbard RA, et al. Assessment and management of cardiovascular risk factors among US veterans with prostate cancer. *JAMA Netw Open*. 2021;4(2):e210070. doi:10.1001/jamanetworkopen.2021.0070

eTable 1. Coding Definitions

eTable 2. Demographic Parameters, Excluded Patients

eFigure. Rates of Comprehensive CVRF Assessment

eTable 3. Unadjusted Proportions and Risk Differences Associated With ASCVD/ADT Status

eTable 4. Sensitivity Analysis: Adjusted Proportions and Risk Differences in Patients With Localized Disease Only

This supplementary material has been provided by the authors to give readers additional information about their work.

© 2021 Sun L et al. *JAMA Network Open.*

eTable 1. Coding Definitions

	System and Codes		
	**ICD-9**	**ICD-10**	**HCPCS (Healthcare Common Procedure Coding System)**
**Androgen Deprivation Therapy**	V07.59	Z79.818	Leuprolide (C9430, J1950, J9217-219)<br>Goserelin (J9202)<br>Abarelix (C9216, J0128, S0165)<br>Triptorelin (J3315)<br>Other (S0175, G9132, J9165, S9560, G0356, J1050, J1051)

**ASCVD**	**ICD-9**	**ICD-10**
Coronary artery disease	410-414, 429,79, 996.03	I20-25, T82.21, T82.85
Stroke	433-438	G45-46, I63, I65, I66, I67, I69
PVD or other atherosclerotic disease	93, 437, 440, 441, 443, 444, 445, 447, 556, V43.4	I70, I71, I73-I75, I77, I79, K55, Z95

**Laboratory Values**	**LOINC (Logical Observation Identifiers Names and Codes)**				
LDL	12773-8	43396-1	91105-7	22748-8	91112-3
	13457-7	46984-1	91106-5	39469-2	91113-1
	18261-8	46985-8	91107-3	56136-5	91114-9
	18262-6	49026-8	91108-1	56137-3	91115-6
	2089-1	49027-6	91109-9	56138-1	91116-4
	2090-9	49132-4	91110-7	56139-9	91117-2
	43392-0	55440-2	91111-5	69419-0	91118-0
	43393-8	57938-3	35198-1	70204-3	
Total cholesterol	12183-0	2565-0	50339-1	9618-0	29765-5
	12531-0	2569-2	56912-9	35200-5	32308-9
	12912-2	48620-9	59038-0	14647-2	39468-4
	2093-3				
HbA1c	41995-2	17855-8	4548-4	4549-2	62388-4
	71875-9	17856-6			
Glucose: fasting or tolerance test	10449-7	1496-9	1526-3	18342-6	26779-9
	10832-4	14995-5	1527-1	20436-2	26780-7
	12610-2	1501-6	1530-5	20437-0	29330-8
	12615-1	1504-0	1533-9	20438-8	32820-3
	12616-9	1506-5	1537-0	20439-6	6689-4
	12617-7	1507-3	1539-6	20440-4	6762-9
	12646-6	1514-9	1540-4	26541-3	10450-5
	12648-2	1517-2	1542-0	26543-9	1554-5
	13606-9	1518-0	1544-6	26777-3	1556-0
	14761-1	1521-4	16914-4	26778-1	1558-6
	1494-4	1525-5	16915-1		

© 2021 Sun L et al. *JAMA Network Open.*

Medications	Medication Class Code (VA National Drug File)
BP-lowering	CV100 (beta blockers), CV200 (calcium channel blockers), CV400 (antihypertensive combinations), CV700 (diuretics), CV800 (ACE inhibitors), CV805 (angiotensin II inhibitors)
Lipid-lowering	CV350
Glucose-lowering	HS500-502, HS509

ASCVD, atherosclerotic cardiovascular disease; PVD, peripheral vascular disease; LDL: low-density lipoprotein

© 2021 Sun L et al. *JAMA Network Open.*

eTable 2. Demographic Parameters, Excluded patients

Characteristic	Excluded: ADT Status Unconfirmed (n=7,257)[a]	Excluded: No PCP Visit (n=2,197)	% Missing, All Excluded (n=9,454)	% Missing, All Included (n=90,494)
Age, median (IQR)	68 (63, 75)	66 (61, 72)	1.7%	0%
Race			7.9%	4.5%
White	4676 (68.7%)	1223 (64.5%)		
African American	1967 (28.9%)	626 (33.0%)		
Other	167 (2.5%)	48 (2.5%)		
Year Category				
2010-2011	1872 (25.8%)	890 (40.5%)		
2012-2013	1912 (26.3%)	569 (25.9%)		
2014-2015	1796 (24.7%)	429 (19.5%)		
2016-2017	1677 (23.1%)	309 (14.1%)		
Number of PCP visits over study period, median (IQR)	5 (3, 8)	. (., .)		
Poverty index above median	4466 (62.2%)	1123 (55.7%)	2.7%	0.7%
Prior ASCVD	1454 (20.0%)	140 (6.4%)		
Smoking Status			31.4%	22.3%
Never	1899 (33.8%)	282 (32.9%)		
Current	1834 (32.6%)	336 (39.2%)		
Former	1892 (33.6%)	240 (28.0%)		
BMI, median (IQR), kg/m^2	28.1 (24.8, 32.1)	27.4 (23.7, 31.1)	23.5%	10.8%
Overweight (BMI≥25 kg/m^2)	4589 (73.8%)	668 (65.9%)		
Obese (BMI>30 kg/m^2)	2273 (36.5%)	327 (32.2%)		
Disease Stage			3.3%	3.3%
Low risk	372 (5.2%)	488 (24.8%)		
Intermediate risk	2037 (28.4%)	511 (26.0%)		
High risk	2840 (39.6%)	623 (31.7%)		
Metastatic or N1	1924 (26.8%)	345 (17.5%)		
PSA			8.6%	5.8%
<10 ng/mL	3269 (47.2%)	1040 (60.6%)		
10-20 ng/mL	1483 (21.4%)	281 (16.4%)		
≥20 ng/mL	2174 (31.4%)	396 (23.1%)		
Gleason Score			12.4%	9.5%
≤6	609 (9.3%)	563 (32.3%)		
7	2783 (42.5%)	685 (39.3%)		
8-10	3149 (48.1%)	493 (28.3%)		
T Stage			18.5%	12.4%
T1-T2a	4006 (65.3%)	1103 (70.3%)		
T2b	321 (5.2%)	43 (2.7%)		
T2c or higher	1812 (29.5%)	424 (27.0%)		

© 2021 Sun L et al. *JAMA Network Open.*

[a]Unless otherwise indicated, data are expressed as number (percentage) of patients with recorded data.

ADT, androgen deprivation therapy; IQR, interquartile range; y, years; PCP, primary care provider; ASCVD, atherosclerotic cardiovascular disease; BMI, body mass index (calculated as weight in kilograms divided by square of height in meters); PSA, prostate specific antigen; T stage, tumor stage.

© 2021 Sun L et al. *JAMA Network Open.*

eFigure. Rates of Comprehensive CVRF Assessment

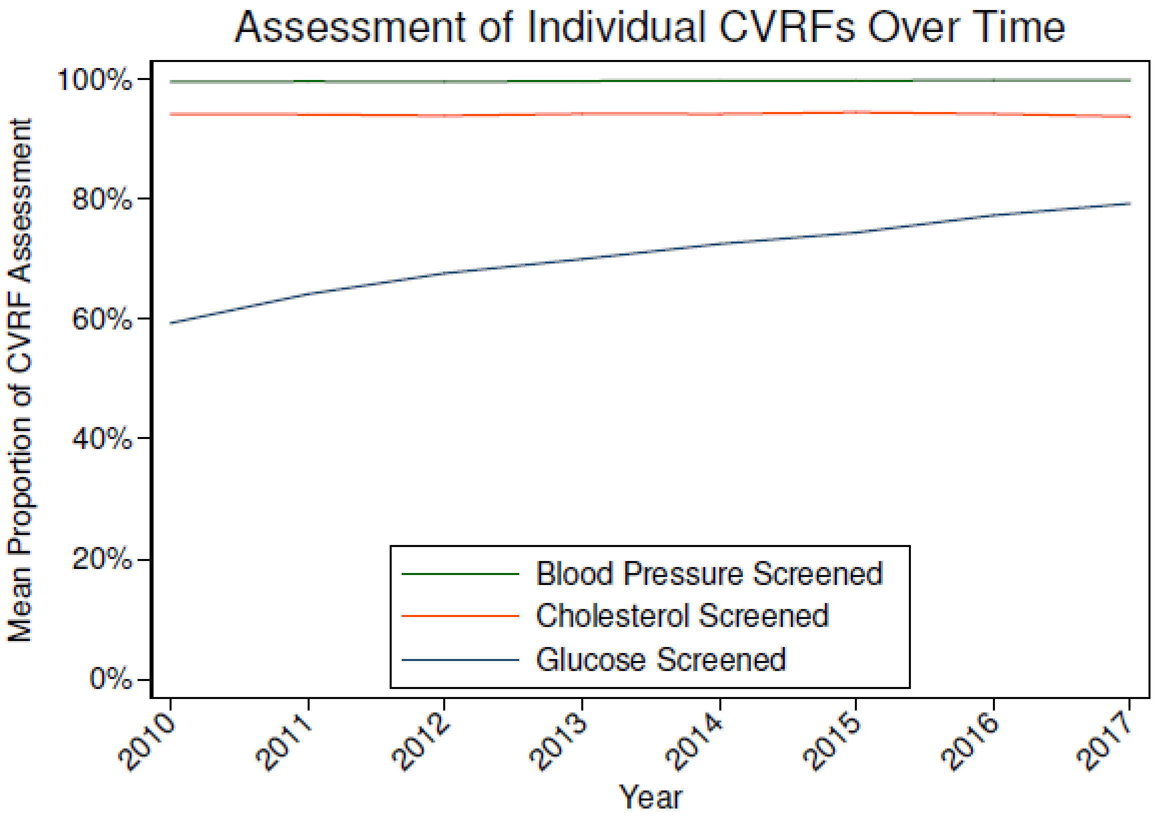

© 2021 Sun L et al. *JAMA Network Open.*

eTable 3. Unadjusted proportions and risk differences associated with ASCVD/ADT status

Comprehensive CVRF Assessment				
	Proportion with Comprehensive Assessment (95% CI), Reference group (ASCVD-/ADT-)[a]	Unadjusted Risk Difference (95% CI)[d]		
		ASCVD+/ADT-	ASCVD+/ADT+	ASCVD-/ADT+
**Comprehensive Assessment (n=90,494)**	66.3% (65.9%,66.7%)	9.7% (8.8%,10.6%)	9.0% (7.7%,10.2%)	0.8% (0.0%,1.5%)
**Uncontrolled CVRF**				
**CVRF (Number with recorded value)**	Proportion Uncontrolled (95% CI), Reference group (ASCVD-/ADT-)[b]	Unadjusted Risk Difference (95% CI)[d]		
		ASCVD+/ADT-	ASCVD+/ADT+	ASCVD-/ADT+
Blood pressure (n=89,572)	35.6% (35.2%,36.0%)	-4.4% (-5.4%,-3.4%)	-3.5% (-4.9%,-2.1%)	3.8% (3.9%,4.6%)
Cholesterol (n=85,243)	22.2% (21.9%,22.6%)	-10.3% (-11.1%,-9.6%)	-11.7% (-12.6%,-10.7%)	-2.8% (-3.5%,-2.1%)
Glucose (n=63,292)	16.8% (16.4%,17.1%)	8.5% (7.5%,9.5%)	10.9% (9.4%,12.4%)	3.2% (2.4%,4.0%)
Any CVRF (n=90,060)	54.3% (53.9%,54.7%)	-4.7% (-5.8%,-3.7%)	-3.1% (-4.6%,-1.6%)	2.3% (1.4%,3.1%)
**Untreated CVRF**				
**CVRF (Number uncontrolled)**	Proportion Untreated (95% CI), Reference group (ASCVD-/ADT-)[c]	Unadjusted Risk Difference (95% CI)[d]		
		ASCVD+/ADT-	ASCVD+/ADT+	ASCVD-/ADT+
Blood pressure (n=31,960)	24.7% (24.1%,25.3%)	-16.0% (-17.1%,-14.8%)	-17.1% (-18.6%,-15.7%)	-4.1% (-5.2%,-3.0%)
Cholesterol (n=16,879)	50.5% (49.6%,51.4%)	-32.1% (-34.5,-29.7%)	-25.2% (-29.2%,-21.2%)	0.4% (-1.6%,2.3%)
Glucose (n=12,098)	8.9% (8.2%,9.6%)	-2.8% (-4.1%,-1.6%)	-1.0% (-2.8%,0.8%)	-1.3% (-2.5%,0.0%)
Any CVRF (n=48,683)	34.1% (33.6%,34.6%)	-22.7% (-23.8,-21.7%)	-21.8% (-23.2%,-20.4%)	-4.6% (-5.6%,-3.5%)

[a] Unadjusted proportion of patients in the reference group (No ASCVD, No ADT) with comprehensive CVRF assessment

[b] Unadjusted proportion of patients in the reference group (No ASCVD, No ADT) with recorded measurements who have uncontrolled CVRFs

[c] Unadjusted proportion of patients in the reference group (No ASCVD, No ADT) with uncontrolled CVRFs who are not on a corresponding risk-reducing medication

[d] Risk difference coefficients are interpreted as absolute percent change in the risk of outcome.

© 2021 Sun L et al. *JAMA Network Open.*

CVRF, cardiovascular risk factor; CI, confidence interval; ADT, androgen deprivation therapy; ASCVD, atherosclerotic cardiovascular disease.

© 2021 Sun L et al. *JAMA Network Open.*

e Table 4. Sensitivity analysis: Adjusted proportions and risk differences in patients with localized disease only

Comprehensive CVRF Assessment				
	Adjusted Proportion with Comprehensive Assessment (95% CI), Reference group (ASCVD-/ADT-)[a]	Adjusted Risk Difference (95% CI)[d]		
		ASCVD+/ADT-	ASCVD+/ADT+	ASCVD-/ADT+
**Comprehensive Assessment (n=81,364)**	66.4% (66.0%,66.8%)	10.3% (9.3%,11.2%)	12.2% (10.7%,13.7%)	3.1% (2.2%,4.1%)
**Uncontrolled CVRF**				
**CVRF (Number with recorded value)**	Adjusted Proportion Uncontrolled (95% CI), Reference group (ASCVD-/ADT-)[b]	Adjusted Risk Difference (95% CI)[d]		
		ASCVD+/ADT-	ASCVD+/ADT+	ASCVD-/ADT+
Blood pressure (n=80,675)	35.9% (35.4%,36.3%)	-4.6% (-5.6%,-3.6%)	-4.2% (-5.9%,-2.5%)	2.8% (1.8%,3.8%)
Cholesterol (n=77,155)	21.8% (21.4%,22.2%)	-9.0% (-9.7%,-8.2%)	-9.4% (-10.6%,-8.3%)	-1.7% (-2.5%,-0.8%)
Glucose (n=57,211)	16.9% (16.5%,17.3%)	8.2% (7.1%,9.3%)	10.8% (9.0%,12.6%)	3.4% (2.5%,4.4%)
Any CVRF (n=81,044)	54.3% (53.9%,54.8%)	-4.1% (-5.2%,-3.0%)	-2.3% (-4.1%,-0.5%)	2.8% (1.8%,3.8%)
**Untreated CVRF**				
**CVRF (Number uncontrolled)**	Adjusted Proportion Untreated (95% CI), Reference group (ASCVD-/ADT-)[c]	Adjusted Risk Difference (95% CI)[d]		
		ASCVD+/ADT-	ASCVD+/ADT+	ASCVD-/ADT+
Blood pressure (n=28,723)	24.3% (23.6%,24.9%)	-16.0% (-17.2%,-14.8%)	-17.2% (-19.0%,-15.4%)	-4.4% (-5.8%,-3.1%)
Cholesterol (n=15,407)	50.2% (49.3%,51.2%)	-32.4% (-34.9,-30.0%)	-30.2% (-34.7%,-25.8%)	-2.9% (-5.2%,-0.5%)
Glucose (n=10,928)	8.9% (8.2%,9.7%)	-3.0% (-4.3%,-1.6%)	-3.6% (-5.5%,-1.6%)	-2.6% (-4.0%,-1.2%)
Any CVRF (n=43,925)	33.7% (33.1%,34.2%)	-22.3% (-23.4,-21.3%)	-23.1% (-24.7%,-21.4%)	-5.7% (-6.9%,-4.5%)

[a] Adjusted proportion of patients in the reference group (No ASCVD, No ADT) with comprehensive CVRF assessment

[b] Adjusted proportion of patients in the reference group (No ASCVD, No ADT) with recorded measurements who have uncontrolled CVRFs

[c] Adjusted proportion of patients in the reference group (No ASCVD, No ADT) with uncontrolled CVRFs who are not on a corresponding risk-reducing medication

[d] Adjusted for race, age, poverty index, disease stage, and year. Risk difference coefficients are interpreted as absolute percent change in the risk of outcome.

© 2021 Sun L et al. *JAMA Network Open.*

CVRF, cardiovascular risk factor; CI, confidence interval; ADT, androgen deprivation therapy; ASCVD, atherosclerotic cardiovascular disease.

© 2021 Sun L et al. *JAMA Network Open.*
